# Magnesium Modifies the Cardiovascular Mortality Risk Associated with Hyperphosphatemia in Patients Undergoing Hemodialysis: A Cohort Study

**DOI:** 10.1371/journal.pone.0116273

**Published:** 2014-12-29

**Authors:** Yusuke Sakaguchi, Naohiko Fujii, Tatsuya Shoji, Terumasa Hayashi, Hiromi Rakugi, Kunitoshi Iseki, Yoshiharu Tsubakihara, Yoshitaka Isaka, the Committee of Renal Data Registry of the Japanese Society for Dialysis Therapy

**Affiliations:** 1 Geriatric Medicine and Nephrology, Osaka University Graduate School of Medicine, 2-2, Yamada-oka, Suita, Osaka, 565-0871, Japan; 2 Committee of Renal Data Registry, Japanese Society for Dialysis Therapy, Tokyo, Japan; 3 Department of Kidney Disease and Hypertension, Osaka General Medical Center, 3-1-56 Bandaihigashi Sumiyoshi-ku, Osaka, 558-8558, Japan; Medical University Innsbruck, Austria

## Abstract

**Background:**

In vitro studies have shown inhibitory effects of magnesium (Mg) on phosphate-induced calcification of vascular smooth muscle cells, raising the possibility that maintaining a high Mg level may be useful for reducing cardiovascular risks of patients with hyperphosphatemia. We examined how serum Mg levels affect the association between serum phosphate levels and the risk of cardiovascular mortality in patients undergoing hemodialysis.

**Methods:**

A nationwide register-based cohort study was conducted using database of the Renal Data Registry of the Japanese Society for Dialysis Therapy in 2009. We identified 142,069 patients receiving in-center hemodialysis whose baseline serum Mg and phosphate levels were available. Study outcomes were one-year cardiovascular and all-cause mortality. Serum Mg levels were categorized into three groups (lower, <2.7 mg/dL; intermediate, ≥2.7, <3.1 mg/dL; and higher, ≥3.1 mg/dL).

**Results:**

During follow-up, 11,401 deaths occurred, out of which 4,751 (41.7%) were ascribed to cardiovascular disease. In multivariable analyses, an increase in serum phosphate levels elevated the risk of cardiovascular mortality in the lower- and intermediate-Mg groups, whereas no significant risk increment was observed in the higher-Mg group. Moreover, among patients with serum phosphate levels of ≥6.0 mg/dL, the cardiovascular mortality risk significantly decreased with increasing serum Mg levels (adjusted odds ratios [95% confidence intervals] of the lower-, intermediate-, and higher-Mg groups were 1.00 (reference), 0.81 [0.66–0.99], and 0.74 [0.56–0.97], respectively.). An interaction between Mg and phosphate on the risk of cardiovascular mortality was statistically significant (*P* = 0.03).

**Conclusion:**

Serum Mg levels significantly modified the mortality risk associated with hyperphosphatemia in patients undergoing hemodialysis.

## Introduction

Cardiovascular disease (CVD) is a leading cause of mortality in patients undergoing hemodialysis, accounting for nearly 40% of all deaths [1]–[4]. Numerous studies have identified risk factors contributing to the excess risk of CVD in this population, such as hypertension, insulin resistance, dyslipidemia, inflammation, oxidative stress, malnutrition, anemia, and uremia [5]. In particular, mineral and bone disorders, especially phosphate retention, are closely involved in the pathogenesis of vascular calcification and CVD in end-stage kidney disease [6]–[8]. Because dietary phosphate restriction and phosphate removal by conventional hemodialysis are often not sufficient to control the serum phosphate level within the optimal range, phosphate binders and/or more frequent hemodialysis are required to improve phosphate control [9], [10].

Magnesium (Mg), the fourth most abundant cation in the human body, plays an essential role in many biological processes. Mg deficiency is known to be involved in various pathological conditions especially CVD [11]. Meta-analyses of community-based cohorts concluded that both a low Mg intake and low serum Mg level are significant risk for CVD events [12]–[14]. The significant association of lower serum Mg with an increased cardiovascular risk is also found in patients with pre-dialysis CKD [15] and those undergoing hemodialysis [16].

Among several mechanisms of the favorable effects of Mg on vascular function, recent studies have focused on its anticalcification property [17], [18]. A cross-sectional study in the general population showed that a higher Mg intake is associated with lower coronary artery calcification scores [19]. Higher serum Mg is associated with a lower prevalence of mitral annular calcification [20] and peripheral arterial calcification [21] in hemodialysis patients. Notably, in vitro studies have shown that Mg can prevent, or even regress, phosphate-induced calcification of vascular smooth muscle cells [22]–[25]. These findings raise the possibility that maintaining a high Mg level attenuates cardiovascular risk associated with hyperphosphatemia. Here, we examined how serum Mg levels affect the association between serum phosphate and cardiovascular mortality risk in patients undergoing hemodialysis

## Materials and Methods

### Data collection

This study used data collected from a database of the Japanese Society for Dialysis Therapy-Renal Data Registry (JRDR). The design and detailed methods of this survey have been described elsewhere [2]. Briefly, the Japanese Society for Dialysis Therapy (JSDT) started annual questionnaire surveys of dialysis facilities throughout Japan in 1968. Since 1983, the JSDT began to prospectively collect patients' information and has been compiling a computer-based registry. The response rate to the questionnaire exceeded 98%, meaning that the database covers nearly all hemodialysis patients in Japan. The study protocol was approved by the Medicine Ethics Committee of the Japanese Society for Dialysis Therapy.

### Study Sample

We used the same dataset as in our previous study (JRDR 11002) [16], which was created from the JRDR database in 2009. The original dataset initially contained 263,849 dialysis patients aged 18 years or older, representing 99% of the dialysis facilities in Japan. We identified all patients who received in-center hemodialysis and whose data on both serum Mg and phosphate levels were available. Patients on peritoneal dialysis, hemofiltration, hemodiafiltration, and short daily or nocturnal hemodialysis were excluded. In Japan, a dialysate Mg concentration of 1.0 mEq/L is prescribed.

### Baseline Covariates

Baseline characteristics were obtained from the database, including age, sex, body mass index (BMI), the primary kidney disease (diabetes or non-diabetes), hemodialysis vintage, duration of hemodialysis treatment (hours per week); laboratory measurements (predialysis serum levels of albumin, urea nitrogen, calcium, phosphate, Mg, C-reactive protein [CRP], hemoglobin, alkaline phosphatase [ALP], and intact parathyroid hormone [PTH]); prescription of phosphate binders (calcium carbonate, sevelamer hydrochloride, and lanthanum carbonate), cinacalcet hydrochloride, and oral and/or intravenous active vitamin D analogue; and history of parathyroidectomy, CVD (myocardial infarction, cerebral infarction, cerebral hemorrhage, and amputation of the extremities), and hip fracture. When the serum albumin level was <4.0 g/dL, the serum calcium level was adjusted as follows: corrected serum calcium level (mg/dL) = measured serum calcium level (mg/dL)+(4.0 – serum albumin level (g/dL)). All laboratory data were based on a single measurement (the latest data as of December 31, 2009).

### Exposure

Serum phosphate level was categorized into quartiles. Serum Mg level was categorized into 3 groups according to our previous study in hemodialysis patients [16], showing that those in the lower-Mg (serum Mg level, <2.7 mg/dL) and higher-Mg (serum Mg level, ≥3.1 mg/dL) groups had a significantly increased mortality risk than those in the intermediate-Mg group (serum Mg level, ≥2.7, <3.1 mg/dL). To confirm that the results were not due to these particular cutoff points, we additionally treated both serum levels of phosphate and Mg as continuous variables and modeled their effects on mortality by using a restricted cubic spline function.

### Outcomes

The study outcomes were 1-year cardiovascular and all-cause mortality, which were obtained from the database in 2010. The classification codes for the underlying causes of deaths have been reported elsewhere [26]. CVD mortality included deaths coded as heart failure, pulmonary edema, ischemic heart disease, arrhythmia, and cerebrovascular disease. The causes of death were ascertained by review of the patients' medical records by the questionnaire respondents.

### Statistical Analysis

Data were presented as the number (percent) for categorical variables and as the mean (standard deviation [SD]) for continuous variables with normal distribution or median [interquartile range] for those with skewed distribution.

Logistic regression models were constructed to calculate odds ratio (OR) and 95% confidence intervals (95%CI) for all-cause and cardiovascular deaths. Multivariable models were adjusted for age; sex; BMI; hemodialysis vintage; duration of hemodialysis treatment; primary kidney disease; serum urea nitrogen, albumin, calcium, Mg, phosphate, ALP, CRP, hemoglobin, and intact PTH level; prescription of phosphate binders, cinacalcet hydrochloride, and active vitamin D analogue; and history of parathyroidectomy, CVD, and hip fracture. We further included a product term between the Mg group (lower and intermediate vs. higher) and the quartile of phosphate (first to third quartiles vs. fourth quartile) in this multivariable model to test for the interaction between magnesium and phosphate on all-cause and cardiovascular mortality. To show the continuous, potentially nonlinear, association of serum phosphate and Mg levels with the risk of mortality, we used adjusted restricted cubic spline models with 4 knots.

All reported P values were 2-sided, and values of *P*<0.05 were considered statistically significant. All statistical analyses were performed using Stata 11.2 statistical software (StataCorp LP, College Station, TX, USA).

## Results

Of the total of 263,849 dialysis patients, those on renal replacement therapy other than in-center hemodialysis (n = 26,752) were excluded. Of the remaining 237,097 patients, both baseline serum Mg and phosphate levels were available in 142,069 patients. We compared all baseline characteristics between those with and without data on serum Mg and phosphate levels, and found no meaningful difference between the groups (Table 1).

**Table 1:** Comparison of baseline characteristics between patients with and without serum magnesium and phosphate level data.

	without data	with data	Total
*Characteristics*	n = 95,028	n = 142,069	n = 237,097
Age, yr	66.2 (12.5)	66.0 (12.5)	66.1 (12.5)
Gender, male, %	62.0	61.9	62.0
HD vintage, yr	7 [4], [11]	7 [4], [12]	7 [4], [12]
HD hour/week	11.6 (1.8)	11.6 (1.7)	11.6 (1.8)
BMI, kg/m2	22.2 (3.8)	22.2 (4.3)	22.2 (4.2)
DM, %	35.8	35.9	35.9
BUN, mg/dL	64.7 (16.7)	64.5 (16.2)	64.6 (16.4)
Adj. Ca, mg/dL	9.3 (0.9)	9.3 (0.9)	9.3 (0.9)
Alb, g/dL	3.7 (0.4)	3.7 (0.4)	3.7 (0.4)
CRP, mg/dL	0.60 (1.84)	0.53 (1.82)	0.55 (1.83)
Hb, g/dL	10.5 (1.3)	10.6 (1.3)	10.6 (1.3)
ALP, IU/L	266 (150)	263 (142)	264 (144)
iPTH, pg/mL	121 [61, 203]	118 [59, 201]	119 [60, 201]
Prescription			
CaCO3, %	58.7	60.9	60.2
Sevelamer, %	26.7	28.4	27.8
Lanthanum, %	13.6	12.9	13.1
Cinacalcet, %	10.2	11.4	11.0
Vit. D (p.o.), %	37.3	38.6	38.2
Vit. D (i.v.), %	25.2	26.6	26.1
Past history			
PTx, %	4.5	5.3	5.1
MI, %	7.5	7.3	7.4
CI, %	14.9	15.0	15.0
CH, %	4.8	4.8	4.8
Amputation, %	3.0	2.9	2.9
Hip fracture, %	2.9	2.9	2.9

Data presented as mean (standard deviation) or median [interquartile range].

Abbreviations: HD, hemodialysis; BMI, body mass index; DM, diabetes mellitus; BUN, blood urea nitrogen; Adj. Ca, albumin-adjusted calcium; Alb, albumin; CRP, C-reactive protein; Hb, hemoglobin; ALP, alkaline phosphatase; iPTH, intact parathyroid hormone; Vit. D, active vitamin D analogue; PTx, parathyroidectomy; MI, myocardial infarction; CI, cerebral infarction; CH, cerebral hemorrhage.

**Figure 1:**
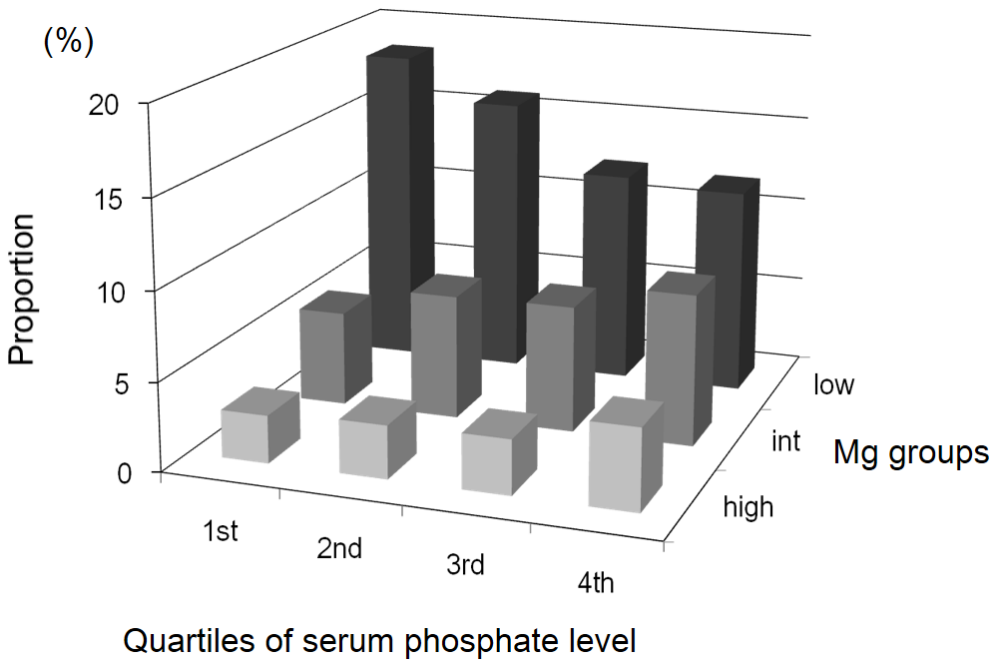
Distribution of the study patients according to quartiles of serum phosphate level and magnesium groups.

Serum levels of Mg and phosphate were normally distributed with a mean value (SD) of 2.6 (0.5) mg/dL and 5.1 (1.5) mg/dL, respectively. Fig. 1 shows the proportion of patients across 3×4 categories of Mg and phosphate. Among the patients in the highest phosphate quartile (serum phosphate levels of ≥6.0 mg/dL), 47.3% were in the lower-Mg group and 34.4% were in the intermediate-Mg group. Baseline characteristics are summarized in Table 2. Intact PTH levels were associated positively with phosphate quartiles (p<0.001 for trend) and negatively with Mg groups (p<0.001 for trend); consequently, those patients in the higher-Mg group of the lowest quartile of phosphate had the lowest median intact PTH level (74 pg/mL).

**Table 2:** Baseline characteristics according to quartiles of serum phosphate levels and magnesium groups.

		Magnesium groups (range of serum magnesium levels [mg/dL])											
		lower (<2.7)				intermediate (≥2.7, <3.1)				higher (≥3.1)			
Quartiles of serum P levels		1st	2nd	3rd	4th	1st	2nd	3rd	4th	1st	2nd	3rd	4th
(range of serum P levels [mg/dL])		(<4.1)	(≥4.1, <5.1)	(≥5.1, <6.0)	(≥6.0)	(<4.1)	(≥4.1, <5.1)	(≥5.1, <6.0)	(≥6.0)	(<4.1)	(≥4.1, <5.1)	(≥5.1, <6.0)	(≥6.0)
*Characteristics*	missing data (%)	n = 26,208	n = 22,681	n = 17,328	n = 16,767	n = 7,618	n = 10,071	n = 10,207	n = 12,181	n = 3,877	n = 4,215	n = 4,421	n = 6,495
Age, yr	0	70.5 (11.4)	68.5 (11.8)	67.4 (11.8)	64.4 (12.5)	66.5 (12.2)	64.4 (12.1)	63.5 (11.8)	60.7(12.5)	67.1 (12.6)	62.9 (12.8)	61.6 (12.2)	58.7 (12.4)
Male, %	0	62.6	62.8	62.4	64.5	59.8	59.6	60.5	63.8	55.6	56.2	59.8	62.5
HD vintage, yr	0	5 [3, 9]	6 [4, 11]	7 [4, 12]	7 [4, 12]	6 [3, 11]	8 [5, 13]	8 [5, 13]	8 [5, 13]	6 [3, 11]	8 [5, 13]	9 [5, 13]	8 [5, 13]
HD, h/wk	0.9	11.3 (2.0)	11.6 (1.8)	11.7 (1.7)	11.7 (1.6)	11.6 (1.7)	11.8 (1.6)	11.8 (1.5)	11.9 (1.5)	11.4 (1.8)	11.8 (1.6)	11.8 (1.5)	11.8 (1.5)
BMI, kg/m^2^	14.0	21.6 (4.8)	22.1 (3.9)	22.4 (4.0)	23.0 (4.9)	21.8 (3.5)	22.0 (4.1)	22.3 (3.5)	22.9 (4.5)	21.3 (5.7)	21.8 (3.5)	22.1 (4.5)	22.7 (4.2)
DM, %	0	40.0	36.4	34.2	34.6	40.7	33.7	31.7	31.5	41.8	35.3	34.6	35.4
BUN, mg/dL	0.1	55.7 (16.4)	59.8 (14.1)	64.1 (13.9)	71.0 (15.4)	63.0 (15.6)	64.7 (13.9)	67.7 (13.6)	73.8(15.1)	63.4 (18.0)	66.7 (14.3)	69.9 (14.2)	76.1 (15.6)
Adj. Ca, mg/dL	1.3	9.26 (0.88)	9.25 (0.81)	9.29 (0.84)	9.27 (0.94)	9.33 (0.84)	9.35 (0.77)	9.38 (0.82)	9.35(0.92)	9.46 (0.97)	9.43 (0.90)	9.45 (0.83)	9.39 (0.88)
Alb, g/dL	1.3	3.5 (0.5)	3.7 (0.4)	3.7 (0.4)	3.7 (0.4)	3.7 (0.4)	3.8 (0.4)	3.8 (0.3)	3.8 (0.3)	3.6 (0.5)	3.9 (0.4)	3.9 (0.4)	3.9 (0.4)
CRP, mg/dL	15.8	0.89 (2.68)	0.54 (1.72)	0.53 (1.79)	0.56 (1.68)	0.50 (1.97)	0.30 (0.95)	0.31 (0.95)	0.34(1.04)	0.69 (2.10)	0.36 (1.33)	0.27 (0.95)	0.29 (1.39)
Hb, g/dL	0.5	10.3 (1.3)	10.5 (1.2)	10.6 (1.2)	10.6 (1.3)	10.6 (1.3)	10.7 (1.2)	10.7 (1.1)	10.8 (1.2)	10.5 (1.4)	10.8 (1.3)	10.9 (1.2)	11.0 (1.3)
ALP, IU	2.3	286 (166)	269 (149)	260 (132)	256 (138)	269 (140)	255 (130)	247 (130)	241 (118)	293 (164)	260 (129)	247 (111)	236 (105)
iPTH, pg/mL	5.2	100 [50, 171]	115 [60, 192]	130 [68, 213]	152 [80, 257]	97 [47, 168]	109 [56, 184]	123 [65, 203]	147 [74, 243]	74 [33, 145]	96 [47, 168]	110 [55, 193]	131 [65, 226]
*Prescription*													
CaCO_3_, %	5.1	53.0	59.7	59.4	59.6	64.9	69.2	67.8	65.6	52.2	63.5	67.7	65.8
Sevelamer, %	5.6	14.0	21.3	26.1	30.4	24.8	33.7	39.3	41.3	23.7	39.7	45.6	49.7
Lanthanum, %	5.8	6.9	9.3	11.8	18.7	11.9	13.3	14.8	20.2	9.9	14.5	15.3	21.4
Cinacalcet, %	6.1	5.7	9.2	12.0	14.2	8.4	12.2	15.1	17.2	7.0	12.8	14.7	17.3
Vit. D (p.o.), %	5.5	38.6	42.2	42.8	38.6	36.0	39.1	38.7	35.6	31.8	34.5	34.6	31.9
Vit. D (i.v.), %	6.1	18.1	24.5	29.0	34.5	20.4	25.7	31.6	34.9	16.7	24.3	29.1	33.0
*Past history*													
PTx, %	9.4	3.5	5.1	6.1	6.5	4.0	6.4	7.0	6.6	3.3	4.8	5.7	5.2
MI, %	10.7	8.7	8.2	8.1	7.9	6.6	5.9	6.0	6.2	6.2	5.6	5.1	5.4
CI, %	10.6	19.0	16.7	15.4	13.8	15.9	12.7	12.1	11.2	20.8	13.3	10.7	9.3
CH, %	10.7	5.3	5.0	4.8	4.3	5.9	4.8	3.8	4.3	7.8	4.9	4.6	3.9
Amputation, %	10.3	3.8	2.7	2.6	3.1	3.3	2.2	2.0	2.6	4.4	2.8	2.2	2.5
Hip fracture, %	10.9	4.4	3.2	2.9	2.5	3.3	2.4	1.8	1.6	3.8	2.4	1.7	1.3

Data are presented as mean (standard deviation) or median [interquartile range].

Abbreviations: P, phosphate; HD, hemodialysis; BMI, body mass index; DM, diabetes mellitus; BUN, blood urea nitrogen; Adj. Ca, albumin-adjusted calcium; P, phosphate; Alb, albumin; CRP, C-reactive protein; Hb, hemoglobin; ALP, alkaline phosphatase; iPTH, intact parathyroid hormone; Vit. D, active vitamin D analogue; PTx, parathyroidectomy; MI, myocardial infarction; CI, cerebral infarction; CH, cerebral hemorrhage.

**Figure 2:**
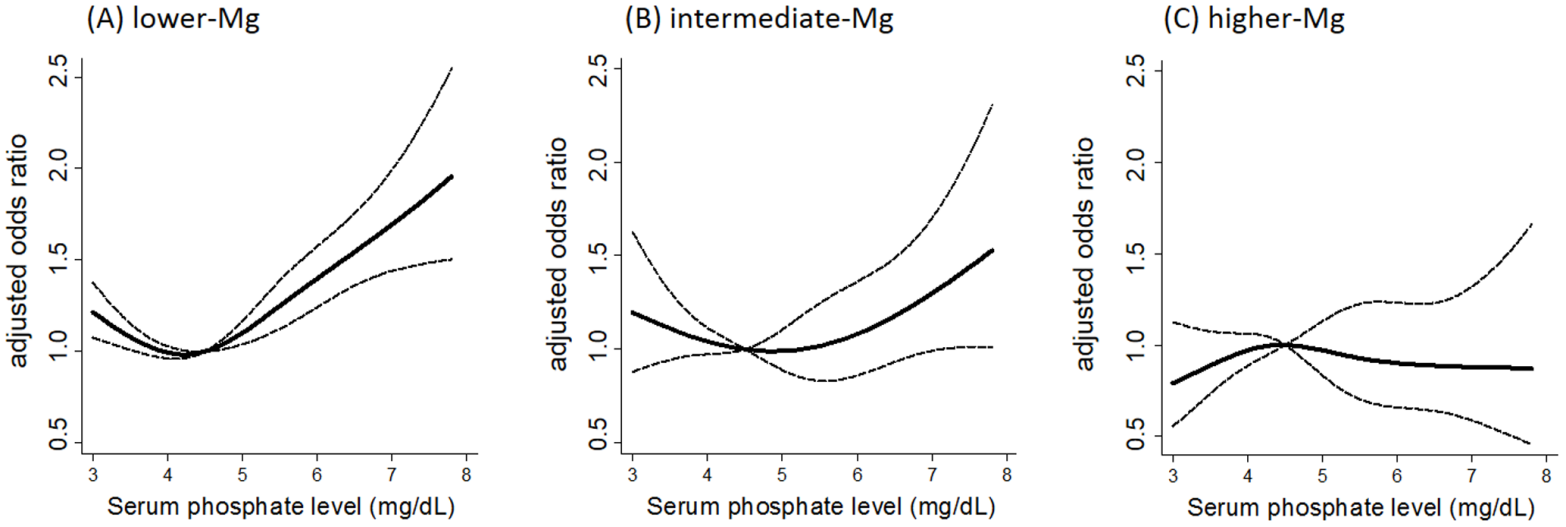
Adjusted odds ratio for cardiovascular mortality. (A) Lower-magnesium group, (B) intermediate-magnesium group, and (C) higher-magnesium group. The dashed line represents the 95% confidence interval. The reference serum phosphate value is at 4.5 mg/dL.

During 1-year follow-up, a total of 11,401 deaths occurred and 4,751 (41.7%) were ascribed to CVD. First, we stratified the patients by the Mg groups and analyzed the association between phosphate and the risk of all-cause and cardiovascular mortality in each Mg group (Tab. 3). In the lower- and intermediate-Mg groups, patients in the fourth phosphate quartile had the highest risk. In contrast, the risk was not significantly elevated with increasing quartiles of phosphate in the higher-Mg group. Restricted cubic spline curves presented similar findings (Fig. 2).

**Table 3:** Adjusted odds ratio for all-cause and cardiovascular mortality according to magnesium groups.

	Magnesium groups								
	lower			intermediate			higher		
*1. all-cause*	OR	95% CI	P	OR	95% CI	P	OR	95% CI	P
*Serum P quartiles*									
1st	1.13	1.04–1.23	0.005	1.23	1.03–1.48	0.02	1.26	0.99–1.62	0.07
2nd	1.00	-	-	1.00	-	-	1.00	-	-
3rd	1.18	1.07–1.30	0.001	1.19	0.98–1.43	0.07	1.14	0.86–1.50	0.36
4th	1.52	1.37–1.69	<0.001	1.58	1.32–1.89	<0.001	1.09	0.83–1.43	0.53
*2. cardiovascular*	OR	95% CI	P	OR	95% CI	P	OR	95% CI	P
*Serum P quartiles*									
1st	1.20	1.06–1.36	0.005	1.20	0.92–1.56	0.17	1.14	0.81–1.61	0.46
2nd	1.00	-	-	1.00	-	-	1.00	-	-
3rd	1.32	1.14–1.52	<0.001	1.10	0.84–1.44	0.49	1.27	0.88–1.84	0.20
4th	1.64	1.41–1.91	<0.001	1.42	1.09–1.84	0.009	1.09	0.75–1.58	0.65

Models were adjusted for age, sex, body mass index, hemodialysis vintage, duration of hemodialysis treatment, diabetes mellitus, serum levels of urea nitrogen, calcium, magnesium, alkaline phosphatase, albumin, C-reactive protein, hemoglobin, and intact parathyroid hormone, prescription of phosphate binders, cinacalcet hydrochloride, active vitamin D analogue, history of parathyroidectomy, CVD (myocardial infarction, cerebral infarction, cerebral hemorrhage, and amputation), and hip fracture.

Abbreviations: P, phosphate; OR, odds ratio; CI, confidence interval; CVD, cardiovascular disease.

**Figure 3:**
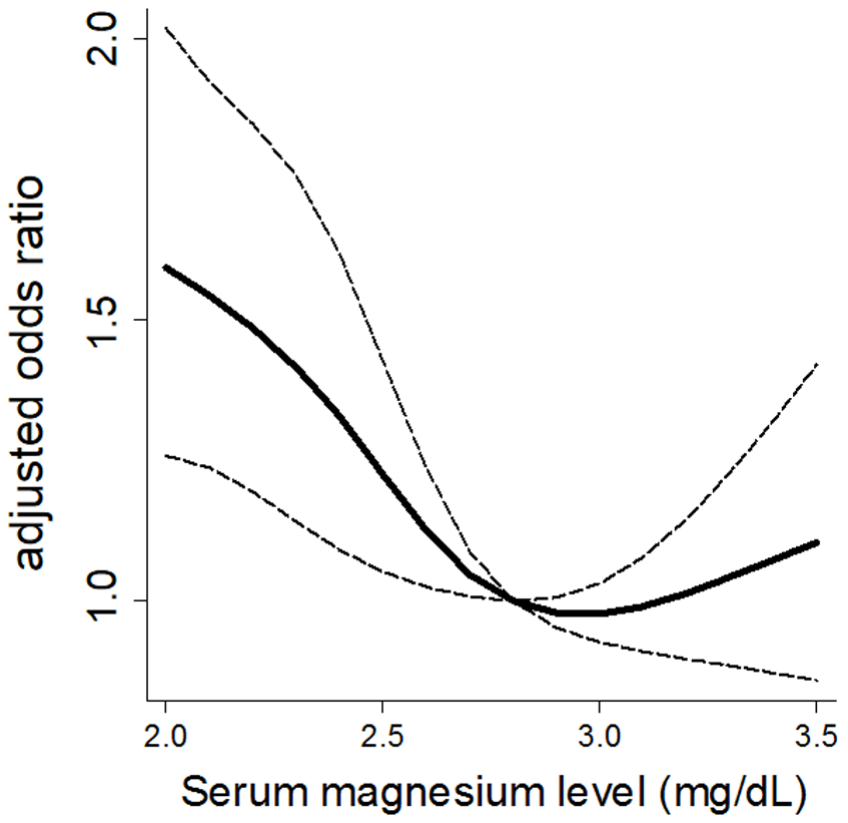
Adjusted odds ratio for cardiovascular mortality in the highest phosphate quartile. The dashed line represents the 95% confidence interval. The reference serum magnesium value is at 2.8 mg/dL.

**Figure 4:**
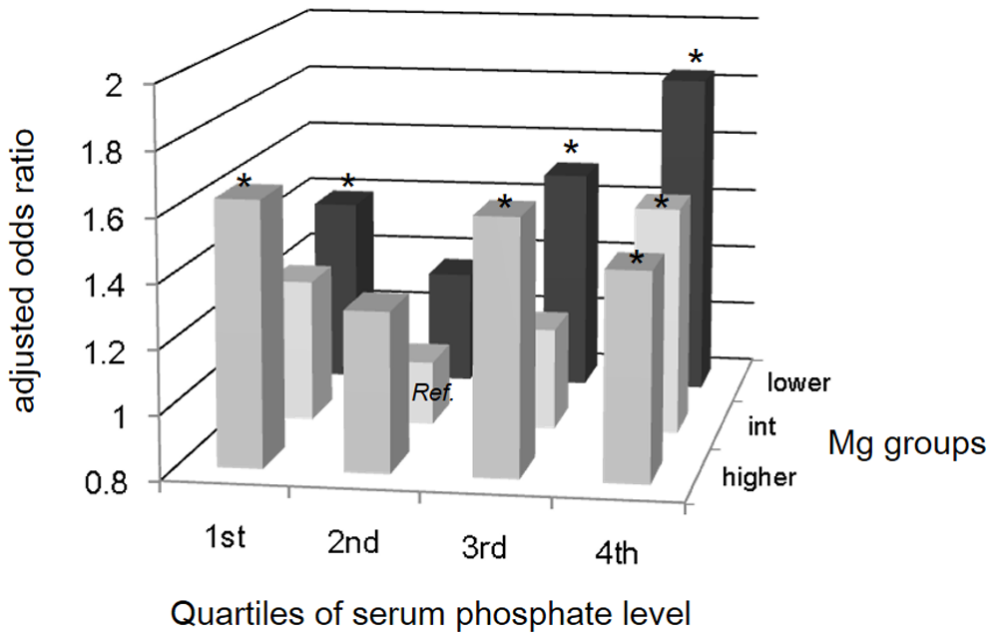
Adjusted odds ratio for cardiovascular mortality across all categories by quartiles of serum phosphate level and magnesium groups. *Ref.*, Reference group; * P<0.05.

Next, the patients were stratified by the quartiles of phosphate, and an association between Mg and the mortality risk was analyzed (Tab. 4). There was a J-shaped association between Mg and mortality risk in the lowest to the third phosphate quartiles. In contrast, the risk monotonously decreased as Mg level increased in the highest phosphate quartile. Restricted cubic spline curves of the association between serum Mg levels as a continuous variable and cardiovascular mortality in the highest phosphate quartile showed a similar trend (Fig. 3). Fig. 4 illustrates the adjusted OR for cardiovascular mortality across all categories by Mg and phosphate. As shown, those patients in the intermediate-Mg group of the second phosphate quartile had the lowest risk, whereas those in the lower-Mg group of the highest phosphate quartile had the highest risk. An interaction term between Mg and phosphate on the all-cause and cardiovascular mortality was statistically significant (*P* = 0.002 and 0.03, respectively).

**Table 4:** Adjusted odds ratio for all-cause and cardiovascular mortality according to quartiles of serum phosphate level.

	Quartiles of serum phosphate											
	Q1			Q2			Q3			Q4		
*1. all-cause*	OR	95% CI	P	OR	95% CI	P	OR	95% CI	P	OR	95% CI	P
*Magnesium groups*												
lower	1.00	-	-	1.00	-	-	1.00	-	-	1.00	-	-
intermediate	0.88	0.77–1.01	0.07	0.78	0.67–0.91	0.002	0.81	0.70–0.95	0.008	0.83	0.73–0.96	0.01
higher	1.17	1.00–1.36	0.05	0.98	0.80–1.21	0.87	0.95	0.77–1.17	0.63	0.71	0.58–0.86	0.001
*2. cardiovascular*	OR	95% CI	P	OR	95% CI	P	OR	95% CI	P	OR	95% CI	P
*Magnesium groups*												
lower	1.00	-	-	1.00	-	-	1.00	-	-	1.00	-	-
intermediate	0.89	0.73–1.07	0.21	0.87	0.70–1.09	0.24	0.76	0.61–0.95	0.02	0.81	0.66–0.99	0.04
higher	1.15	0.93–1.42	0.19	1.14	0.85–1.53	0.37	1.08	0.82–1.43	0.60	0.74	0.56–0.97	0.03

Models were adjusted for age, sex, body mass index, hemodialysis vintage, duration of hemodialysis treatment, diabetes mellitus, serum levels of urea nitrogen, calcium, phosphate, alkaline phosphatase, albumin, C-reactive protein, hemoglobin, and intact parathyroid hormone, prescription of phosphate binders, cinacalcet hydrochloride, active vitamin D analogue, history of parathyroidectomy, CVD (myocardial infarction, cerebral infarction, cerebral hemorrhage, and amputation), and hip fracture.

Abbreviations: OR, odds ratio; CI, confidence interval; CVD, cardiovascular disease.

We performed two sensitivity analyses. First, although data on ionized Mg levels were not available in this study, we repeated the analyses by using albumin-adjusted serum Mg levels calculated from the following formula: albumin-adjusted serum Mg (mmol/L) = serum Mg (mmol/L)+0.05 (4.0 – serum albumin [mg/dL]), if serum albumin is ≤4.0 mg/dL [27]. Similar findings were obtained in this analyses; among patients with serum phosphate levels of ≥6.0 mg/dL, the adjusted ORs (95% CI) for cardiovascular mortality of the lower-, intermediate-, and higher-Mg groups were 1.00, 0.75 (0.59–0.96), and 0.77 (0.60–0.97), respectively.

Second, although there is no specific definition of “hypomagnesemia” in patients undergoing hemodialysis, the range of serum Mg level in the lower-Mg group actually consisted of low, normal, and high levels of serum Mg, which might bias the results. Therefore, we divided the patients in the lower-Mg group into two subgroups: very low-Mg (serum Mg level <2.0 mg/dL) and lower-Mg (serum Mg level ≥2.0, <2.7 mg/dL). Because the proportion of patients whose serum Mg level was below the reference range (<1.8 mg/dL) was only 1.4% of the total cohort, the cutoff point between the very low- and lower-Mg groups was based on the lower fifth percentile of serum Mg level. Among patients with serum phosphate levels of ≥6.0 mg/dL, the risk for cardiovascular mortality was not significantly different between the very low- and lower-Mg groups (adjusted ORs [95% CI] for cardiovascular mortality of the very low-, lower-, intermediate- and higher-Mg group were 1.21 [0.81–1.82], 1.00, 0.82 [0.67–1.01], and 0.76 [0.57–1.00]), respectively.

## Discussion

In this large-scale cohort of hemodialysis patients, we demonstrated that the mortality risk associated with hyperphosphatemia was largely altered by serum Mg levels. In contrast to the previous studies examining the association of either serum Mg or phosphate alone with mortality [6], [7], [16], the novelty of the current study is that we evaluated the combined effect of these two minerals on the risk of cardiovascular mortality. In accordance with the recent in vitro studies showing the protective role of Mg on phosphate-induced calcifications of VSMCs [22]–[25], we found that the mortality risk of patients with hyperphosphatemia was significantly attenuated with increasing serum Mg levels. This finding suggests that, in addition to the current strategies to reduce phosphate load, increasing Mg levels may also help to attenuate the cardiovascular risk of patients with hyperphosphatemia.

Several ways to increase serum Mg levels can be considered. As one of the major determinants of serum Mg in hemodialysis patients is a dialysate Mg concentration, increasing the dialysate Mg level is a simple way to increase serum Mg. Most patients in our study were dialyzed with the dialysate Mg level of 1.0 mEq/L, and approximately 80% of patients with hyperphosphatemia were in the lower- and intermediate-Mg groups. Therefore, a higher dialysate Mg concentration may be beneficial for these patients, which should be examined by further studies.

Another determinant of serum Mg is the daily amount of Mg intake [28]. Although it would be important for those without advanced kidney failure to increase their dietary Mg intake which may be protective for the cardiovascular risk [12]–[14], it seems impractical for patients with end-stage kidney disease, who need to restrict potassium intake, to increase their dietary Mg intake because Mg-rich foods are often abundant sources of potassium [28]. In this regard, Mg-containing phosphate binders which can increase purely serum magnesium levels may be a valuable treatment option for hyperphosphatemia of hemodialysis patients.

It should be noted that, in hemodialysis patients who are unable to excrete Mg from the kidney, increasing Mg intake may lead to an excess amount of body Mg which has adverse clinical consequences, especially abnormality of bone metabolism. Although the role of Mg on uremic bone disorder is currently not completely understood, a previous bone biopsy study in 100 patients undergoing hemodialysis found no association between bone Mg content and osteomalacia or any other type of bone disease [29]. Recently, Covic A et al. reported that the use of Mg-containing phosphate binders did not have a distinct effect on bone turnover markers [30]; thus, the authors stated that the drug causes neither an overstimulation nor a suppression of bone turnover. Although the study supports that the drug has a minimal risk on bone metabolism, long-term follow-up studies are needed to confirm its safety.

We unexpectedly found that patients in the higher-Mg group of the lowest phosphate quartile had the second highest risk. This may be owing to the poor baseline clinical characteristics in this patient group, such as a high proportion of prior history of cerebral infarction, cerebral hemorrhage, and amputation of the extremities. In addition, the patients in this group had very low levels of PTH, which is known to be associated with a high mortality risk [31]. The high Mg and low phosphate as well as a slightly high calcium levels in this category are considered to contribute to the oversuppression of PTH.

Several limitations should be noted in the interpretation of our findings. First, the observational nature of the study design cannot infer causality. The short follow-up period might increase the risk of reverse causality. We cannot exclude residual confounding as a potential explanation for Mg being a significant effect modifier of the association between phosphate and the mortality risk. For example, blood pressure level, lipid profile, vitamin D level, smoking status, and type of vascular access were not available and are not accounted for in the multivariable models. In addition, a lower serum Mg level has been linked to older age, malnutrition, inflammation, and cardiovascular comorbidities that might not be fully accounted for in the multivariable analyses despite the extensive adjustment for these factors. However, it should be noticed that those patients who were the oldest, had the highest CRP and the lowest albumin levels were in the lower Mg-lowest phosphate quartile group, but not in the lower Mg-highest phosphate quartile (the highest risk group); therefore, it is unlikely that the high mortality risk observed in the latter group was merely the reflection of malnutrition and inflammation. Second, there are some drawbacks in the data collection. Several baseline variables contained missing values, although most represented 10% or less. The method of data collection, including the ascertainment of the causes of death, was not standardized. All laboratory data were based on a single measurement. These problems arose from the questionnaire-based surveillance of the very large sample size, which is, in turn, a major strength of this study. Finally, there is a considerable international difference in the clinical practice and prognosis of patients undergoing hemodialysis. Because the target range of intact PTH level in Japan during the study period was set at 60–180 pg/mL, which was lower than that in the K/DOQI (Kidney Disease Outcomes Quality Initiative) guidelines (150–300 pg/mL), patients in our cohort might be more prone to be affected by the low intact PTH level. The external validity of our findings to the non-Japanese population should be examined.

Despite these limitations, we conclude that the mortality risk associated with hyperphosphatemia is significantly modified by serum Mg levels in hemodialysis patients; the risk decreases as serum Mg levels increase. In addition to the conventional therapeutic approach to phosphate load, maintaining a high Mg level may also be beneficial to improve cardiovascular prognosis of hemodialysis patients with hyperphosphatemia.
